# Understanding cardiovascular injury after treatment for cancer: an overview of current uses and future directions of cardiovascular magnetic resonance

**DOI:** 10.1186/1532-429X-15-66

**Published:** 2013-07-31

**Authors:** Sujethra Vasu, W Gregory Hundley

**Affiliations:** 1 Department of Internal Medicine (Section on Cardiology), Wake Forest School of Medicine, Winston-Salem, North Carolina, USA; 2 DepartmentRadiology, Wake Forest School of Medicine, Winston-Salem, North Carolina, USA

**Keywords:** Cardiotoxicity, Chemotherapy, Cancer, Cardiovascular magnetic resonance

## Abstract

While cancer-free survival has improved over the past 20 years for many individuals with prostate, renal, breast, and hematologic malignancies, the increasingly recognized prevalence of cardiovascular (CV) events in cancer survivors has been an unintended consequence of many of the therapies that have improved these survival rates. The increase in CV events threatens to offset the improvement in cancer related survival. As a result, there is an emerging need to develop methods to identify those individuals treated for cancer at increased risk of cardiovascular events. With its inherent ability to characterize myocardial tissue and identify both cardiac and vascular dysfunction, cardiovascular magnetic resonance (CMR) has the potential to identify both subclinical and early clinical CV injury before the development of an overt catastrophic event such as a myocardial infarction, stroke, or premature cardiac death. Early identification provides an opportunity for the implementation of primary prevention strategies to prevent such events, thereby improving overall cancer survivorship and quality of life. This article reviews the etiology of CV events associated with cancer therapy and the unique potential of CMR to provide early diagnosis of subclinical CV injury related to the administration of these therapies.

## Review

Over the past 20 years, cancer free survival has improved for many individuals with prostate, renal, breast, and hematologic malignancies. Unfortunately, an unintended consequence of many of the therapies that have contributed to this improvement in cancer-free survival has been the increasingly recognized evolution of cardiovascular (CV) events [1-12]. Several recently published research studies provide insight into possible etiologies of these events. Result from studies involving the Centers for Medicare-Medicaid Services (CMS) and Health Maintenance Organization (HMO) databases within the United States indicate an increased prevalence of billing codes for heart failure, myocardial infarction (MI), and cardiac arrhythmias in patients treated for cancer [1-12]. Also in these studies, there is an increased frequency of codes related to the administration of chemotherapy for cancer that precedes the onset of codes for CV disease and events. The fact that chemotherapy codes precede CV event codes in cancer patients implies a temporal relationship between the administration of cancer treatment and the subsequent occurrence of CV events.

Importantly, the number of cancer survivors who experience subsequent CV events is large. In the US alone, there are now over 13 million cancer survivors, and for breast cancer alone, it is estimated that over $800 million will be spent annually providing CV care for these women [2-4]. As a result, there is an emerging need to develop accurate, cost-effective methods to identify those individuals treated for cancer at increased risk of CV events.

Cardiovascular magnetic resonance (CMR) with its inherent ability to characterize myocardial tissue and identify both cardiac and vascular dysfunction has the potential to identify both subclinical and early clinical CV injury before the development of an overt catastrophic event such as a MI, stroke or premature cardiac death. Early identification of subclinical CV injury provides an opportunity for the implementation of primary prevention strategies to prevent these untoward CV events. By reducing CV related events in those treated for cancer, the opportunity exists to improve overall cancer survivorship.

In this article, we review the data indicating an increased incidence of CV events in cancer survivors, the underlying mechanisms of these events, and the potential of CMR to provide early diagnosis of subclinical CV injury related to the administration of therapy for cancer, and therefore to guide therapeutic interventions to reduce the overall CV related mortality and morbidity associated with therapy for cancer.

### Cardiovascular injury and disease after cancer treatment

The 5-year survival rate for patients with breast cancer or hematologic malignancies has increased from an average of 53% in 2007, to upwards of 85% in 2012 [13,14]. While encouraging, this positive trend in improved cancer-related mortality is tempered by an emerging increase in CV disease, morbidity, and mortality [8]. The reasons for this increase in CV related events are uncertain; however, the results from several studies suggest that this emergence may be related to the therapies utilized to treat the cancer. Several therapeutic interventions including the administration of chemotherapy [15], immunotherapy [16], hormone deprivation [17] and radiation related therapy [18,19] have been associated with CV related increases in morbidity or mortality. The injuries and abnormalities associated with cancer treatment can be highly variable and depend on the type of cancer treatment received. In Table 1, a review of the agents previously associated with CV injury is provided. Below, we briefly discuss three categories of agents associated with CV events.

**Table 1:** Cancer therapeutic agents, risk factors, mechanisms, and manifestations of cardiotoxicity

**Therapeutic agent**	**Therapeutic indications**	**Risk factors**	**Mechanisms**	**Manifestations of cardiotoxicity**
**Anthracyclines**				
Doxorubicin	Breast cancer	Concurrent chemotherapy	Cellular apoptosis induction	*Early*
Daunorubicin	Gastric	Dosing schedules	ETC. uncoupling	CHF/LV dysfunction
Epirubicin	Leukemias	Elderly	Iron complexation	Myocardial ischemia/infarction
Idarubicin	Lung cancer	Women	Lipid peroxidation of myocyte	Pericarditis/myocarditis
	Lymphomas	Prior radiation	membranes	QT prolongation
	Ovarian	IV administration	Nuclear DNA damage	ST-T wave abnormalities
	Sarcomas	Underlying CV disease	ROS formation	*Late*
				Cardiomyopathy
				CHF/LV dysfunction
**Anthraquinolones**				
Mitoxantrone	AML	Unknown	ROS formation	Arrhythmias
				CHF
	Breast cancer			Myocardial ischemia/infarction
	NHL			
**Antimetabolites**				
5-Fluorouracil	Breast cancer	Underlying CV disease	Endothelial cell damage	Arrhythmias
	Colorectal cancer		Vasospasm	CHF
	Pancreatic cancer			Myocardial ischemia/infarction
**Antimicrotubules**				
Paclitaxel	Breast cancer	Unknown	Hypersensitivity reaction	Bradyarrhythmias
				CHF
	Kaposi’s sarcoma			Hypotension
				Myocardial ischemia/infarction
	Lung cancer			
	Ovarian cancer			
Vinca alkaloids	Leukemias	Unknown	Possible vasospasm	Autonomic neuropathy
Vinblastine	Lymphomas			Hypotension
Vincristine	Nephroblastoma			Myocardial ischemia/infarction
				Raynaud’s phenomenon
**Alkylating agents**				
Busulfan	CML	Unknown	Unknown	Arrhythmias
				Pericardial effusion
				HTN
				Pulmonary fibrosis
Cisplatin	Germ cell tumors	Elderly	Coronary artery fibrosis	*Early*
	Lung cancer	Prior mediastinal irradiation	Hypokalemia	CHF
	Lymphomas	Use for metastatic testicular	Hypomagnesaemia	Myocardial ischemia/infarction
	Ovarian cancer	cancer		*Late*
	Sarcomas	Use with cyclophosphamide		Arrhythmias
				HTN
				LVH
				Myocardial ischemia/infarction
Cyclophosphamide	Leukemias	High dose regimens	Endothelial capillary damage	CHF/LV dysfunction
	Lymphomas	Prior mediastinal irradiation		Hemorrhagic myocardial necrosis
	Various solid tumors	Prior anthracyclines		Hemorrhagic pericarditis
				LVH
Ifosfamide	Lymphomas	High dose regimens	Myocardial fiber fragmentation	Arrhythmias
	Various solid tumors	Use for lymphomas		CHF
**Biological agents**				
Interferon-α	Leukemias		Unknown	*Early*
	Lymphomas			Arrhythmias
	Melanoma			Hypertension
	Various solid tumors			*Late*
				Cardiomyopathy
Interleukin-2	Melanoma		Unknown	*Early*
	RCC			Hypotension (capillary leak syndrome)
				Myocarditis
				Thrombotic events
				Ventricular arrhythmias
				*Late*
				Dilated Cardiomyopathy
**Hormone-modifying therapy**				
Androgen-deprivation therapy	Prostate cancer	Men over 65	Development of metabolic	CAD
		Underlying CV disease	syndrome	CHF/LV dysfunction
			Dyslipidemia	Myocardial ischemia/infarction
			Insulin resistance	QT prolongation
			Obesity	SCD
Aromatase Inhibitors	Breast cancers	Unknown	Dyslipidemia	CAD
	Estrogen receptor (+)			CHF/LV dysfunction
				Myocardial ischemia/infarction
**Miscellaneous**				
All-trans retinoic acid (Tretinoin)	APL	Unknown	Unknown	Arrhythmias
				CHF
				Hypotension
				Myocardial ischemia/infarction
				Pericardial effusions
Arsenic trioxide	AML	Unknown	Hypomagnesaemia	Arrhythmias with QT prolongation
				Pericardial effusion
Pentostatin	Hairy cell leukemia	Use with cyclophosphamide	Unknown	Arrhythmias including A-V block
				CHF
				Myocardial ischemia/infarction
**Radiation therapy**				
	Various malignancies	Prior high doses of radiation	Fibrosis caused by inflammatory	*Early*
		Underlying CV disease	changes	Pericarditis/Pericardial effusion
		Use with anthracyclines	ROS formation	*Late*
				CAD
				CHF
				Conduction abnormalities
				Constrictive pericarditis
				Restroctive cardiomyopathy
				Valvular defects
**Tyrosine-kinase inhibitors**				
Bevacizumab	Colorectal cancer	Use with anthracyclines	Monoclonal antibody against VEGF	Arterial and venous thromboembolism
	HER-2 (−) breast cancer		Possible decrease in nitric oxide and	CHF
	Lung cancer		prostaglandin production	HTN
Imatinib	GIST	Use with anthracyclines	Unclear, but may induce apoptosis in cardiomyocytes	CHF/LV dysfunction
	Leukemias			Pericardial effusion
Lapatinib	HER-2 (+) breast cancer	Use with anthracyclines	Inhibits HER-2 and EGFR	LV dysfunction
				QT prolongation
Sorafenib	HCC	Use with anthracyclines	Unclear, but may induce apoptosis in cardiomyocytes, or inhibit VEGF and RAF-1	CHF/LV dysfunction
	RCC			HTN
				Myocardial ischemia/infarction
				Thromboembolism
				CHF/LV dysfunction
Sunitinib	GIST	Use with anthracyclines	Unclear, but may induce apoptosis in cardiomyocytes and inhibit VEGF	HTN
	RCC			Thrombotic events
Trastuzumab	HER-2 (+) breast cancer	Elderly	Defects in HER-2 signaling associated with cardiac contractility	*Early*
		Prior mediastinal irradiation		CHF
		Underlying CV disease	Immune-mediated destruction of cardiomyoytes caused by selective binding to HER-2 protein	LV dysfunction
		Use with anthracyclines		*Late*
				Cardiomyopathy

*AML* acute myeloid leukemia, *APL* acute promyelocytic leukemia, *CAD* coronary artery disease, *CHF* congestive heart failure, *CML* chronic myeloid leukemia, *CV* cardiovascular, *EGFR* epidermal growth factor receptor, *ETC*. electron transport chain, *GIST* gastrointestinal stromal tumor, *HCC* haptocellular carcinoma, *HER* human epidermal growth factor receptor 2, *HTN* hypertension, *LV* left ventricle, *LVH* left ventricular hypertrophy, *NHL* non-Hodgkin’s lymphoma, *RCC* renal cell carcinoma, *ROS* reactive oxygen species, *SCD* sudden cardiac death, *VEGF* vascular endothelial growth factor.

#### Myocellular injury due to anthracycline chemotherapy

In children or adults treated for a hematologic malignancy or women treated for breast cancer, the administration of anthracyclines has been associated with left ventricular (LV) dysfunction and heart failure [9,20]. In the Childhood Cancer Survivor Study (CCSS) cohort, which includes children and young adults <21 years of age who received chemotherapy beginning in 1994, childhood cancer survivors experienced a relative risk (RR) of 15.1 (95% confidence interval [CI] 4.8-47.9) of developing congestive heart failure (CHF) when compared to their siblings without cancer or receipt of chemotherapy [20]. In a separate study of 31,748 women diagnosed with breast cancer, anthracycline chemotherapy was associated with the development of cardiomyopathy (hazard ratio [HR] 2.48, 95% CI 2.1 -2.93) and CHF (HR 1.38, 95% CI 1.25-1.52) [21].

In addition to CHF, patients exposed to anthracyclines experience other CV events including MI and stroke. In the CCSS cohort, children treated with anthracyclines developed coronary artery disease (CAD; RR 10.4, 95% CI 4.1-25.9) and cerebrovascular accidents (RR 9.3, 95% CI 4.1-21.1) more frequently than their siblings without cancer [22-26]. Similarly, in women over the age of 65 years treated with adjuvant anthracycline chemotherapy for Stage I or II breast cancer, MI, stroke, and other CV events were the primary cause of death in those surviving 5 years beyond initiation of their treatment [8].

In adults, the combination of anthracyclines and radiation therapy further increases CV events, particularly MI. In the British National Lymphoma Investigation database, a higher incidence of MI was noted in survivors of Hodgkin’s disease who received radiation therapy and anthracycline-based chemotherapy [27]. The RR of death due to MI after radiation therapy ranged from 1.6 to 9.5 depending on radiation type and the associated chemotherapy regimen utilized in the treatment plan [27]. Furthermore, the RR of death due to MI was 4.1 in the first year after treatment and remained elevated at 2.5 for more than 25 years after treatment.

Similar late risks of cardiac death were noted in 1,080 patients with Hodgkin’s lymphoma aged ≤50 years. In these patients, radiation and chemotherapy were associated with cardiac mortality (RR 3.2 [1.9-5.2]) that remained elevated (RR 4.5 [1.2-11.6]) 20 years after treatment [28]. Overall, as described by Yeh et al., the cumulative dose of anthracycline, the concomitant administration of other cardio-toxic agents, prior radiation therapy, female gender, increasing age, or the presence of diabetes or hypertension are risk factors for developing cardiovascular injury upon receipt of anthracycline based chemotherapy with or without radiation therapy [29-33].

#### Tyrosine kinase inhibitors - trastuzumab

Tyrosine kinases modulate cellular growth, differentiation and metabolism [34]. Inhibitors of tyrosine kinases have been associated with down regulation of many cancer cell related functions [35]. In general, these agents are of two broad types: monoclonal antibodies, such as trastuzumab and bevacizumab, and small molecule inhibitors, such as lapatinib, imatinib, sorafenib, and sunitinib. Over the past 5 to 10 years, it has been recognized that many of these agents are also associated with several adverse CV related abnormalities including microvascular injury, hypertension, and LV dysfunction [35,36]. The administration of the tyrosine kinase inhibitor trastuzumab to women with HER2-positive breast cancer has been associated with subclinical deteriorations in left ventricular ejection fraction (LVEF) [36]. Piccart-Gebhart, et al. identified the incidence of clinical CHF or a subclinical deterioration of LVEF, defined as a decrease in LVEF by 10% [37], to be 1.7% and 7%, respectively, in women receiving trastuzumab. In a separate study by Seidman, et al. cardiac dysfunction, defined as symptomatic CHF or an asymptomatic deterioration in LVEF of 10%, occurred in 27% of women who received trastuzumab versus 8% receiving an anthracycline/cyclophosphamide combination [16]. Cardiac dysfunction with trastuzumab has been previously reported to occur in 13% of women as opposed to only 1% of women who received paclitaxel without trastuzumab [16].

Anthracycline cardiotoxicity appears to be potentiated by the concomitant administration of trastuzumab. This particular combination is associated with the development of severe LV systolic dysfunction, and as a result, these agents are now administered in series (as opposed to simultaneously) when utilized to treat breast cancer. Furthermore, some trials have shown that increasing the duration between anthracycline and trastuzumab administration reduces the incidence of LV dysfunction during treatment for breast cancer [16,38].

#### Hormone deprivation therapies

In women with breast cancer or men with prostate cancer, the application of hormone deprivation therapies (aromatase inhibitors for post-menopausal women and gonadotropin releasing hormone [GnRH] agonists, anti-androgens, or orchiectomy in men) have dramatically improved cancer-related survival and reduced cancer-related recurrence [39-42]. Importantly however, it is increasingly recognized that these therapies are associated with CV events [43,44]. Androgen deprivation therapy (ADT) is associated with the development of peripheral arterial disease (PAD) and cerebrovascular disease including stroke or transient ischemic attack (TIA) [45]. Among 182,757 men >66 years in age, ADT was associated with a 5-year increased incidence of PAD upon receipt of GnRH agonists (adjusted HR 1.16, 95% CI 1.12–1.21) or after bilateral orchiectomy (adjusted HR 1.13, 95% CI 1.02–1.26) [45]. Both therapeutic strategies were associated with an increase in venous thromboembolism (adjusted HR 1.10, 95% CI 1.04–1.15; and adjusted HR 1.27, 95% CI 1.11–1.45, respectively) [45]. Prior studies have reported that PAD develops shortly (as early as 1 to 4 months) after ADT initiation and is often accompanied by an increase in incident diabetes and serum lipoprotein abnormalities.

Multiple studies have confirmed an increased risk of stroke/TIA after receipt of GnRH agonists, oral anti-androgens or bilateral orchiectomy [46,47]. Among 22,310 men with prostate cancer followed for an average of 3.9 years, ADT use was associated with the development of stroke/TIA, specifically GnRH agonists (adjusted RR 1.18, 95% CI 1.00–1.39), oral anti-androgens (adjusted RR 1.47, 95% CI 1.08–2.01), and those who underwent bilateral orchiectomy (adjusted RR 1.77, 95% CI 1.25– 2.39) [48]. The risk was not modified based on the presence of underlying cardiac risk factors with the highest risk seen in men <65 years of age (adjusted HR 2.47, 95% CI 1.24-4.47).

In post-menopausal women, the administration of aromatase inhibitors reduces breast cancer recurrence [42,49]. The etiology of this reduction is felt to be related to the severe reduction in circulating estrogens to near unmeasurable levels [40]. It is becoming increasingly recognized, however, that vascular disease and associated CV events are becoming more prevalent upon receipt of aromatase inhibitors. In a meta-analysis of seven trials that included 30,023 patients comparing aromatase inhibitors and tamoxifen, longer durations of aromatase inhibitor use was associated with increases in the odds ratios of developing CV disease (OR 1.26, 95% CI 1.10-1.43, p < 0.001) [39].

In summary, for some individuals, traditional and newer therapeutic interventions for cancer (Table 1) can promote a variety of both cardiac (CHF) and vascular (MI, stroke, PAD) associated disorders. With the emergence of these disorders affecting multiple components of the CV system, there exists a unique opportunity to utilize imaging to both diagnose and guide therapeutic interventions to diagnose and then guide the administration of therapeutic interventions to prevent these untoward effects.

### Identification of cardiac toxicity

For the heart, several strategies have been utilized to identify myocardial injury related to the administration for cancer [50]. Upon the first recognition of anthracycline-related myocardial dysfunction, endomyocardial biopsies were performed to identify histopathologic evidence of myofibrillar loss, vacuolization and extracellular loss [51,52] and thereby confirm or refute the presence of cardiotoxicity. Importantly however, this form of monitoring became impractical for widespread clinical application as the technique required an invasive procedure that was not well-suited for repetitive examinations [50].

In the 1970s and 1980s, serial multigated nuclear radioisotope studies or MUGA exams were serially implemented to detect LV systolic dysfunction in patients treated with anthracycline-based chemotherapy [53,54]. Evidence of deterioration in LVEF on MUGA scans during receipt of anthracycline chemotherapy was associated with the development of CHF [53,55]. Today, many of the existing management protocols rely on serial measures of LVEF by MUGA or transthoracic echocardiography to identify chemotherapy or immunotherapy induced reductions in LV ejection fraction [56,57]. Importantly however, distinguishing relatively small changes in LVEF related to cardiotoxicity from those related to variance in the technique can be problematic. As a result, more advanced transthoracic echocardiography (strain or diastolic function) assessments, [58] with or without concomitant serum biomarkers (such as serum troponin I levels [59,60]) have been utilized with a goal of increasing detection of early evidence of cardiac injury after chemotherapy [61,62]. The goal of these strategies is to identify those at risk of future CV events in order to implement therapy to prevent these events.

It is important to note, however, that difficulties remain with some of these newer quantitative strategies. Echocardiography can be difficult to reliably perform longitudinally over time due to body habitus, prior radiation treatment, or prosthetic implants, and therefore, it can be difficult to obtain adequate images from reproducible slice positions in a reliable fashion. While serum biomarkers can be easily acquired, these tests can lack specificity for cardiac injury related to the administration of therapy for cancer. Reduced specificity of identification of chemotherapy-related cardiac injury can lead to the unnecessary premature termination of chemotherapy that prevents an individual cancer patient from realizing the full benefit of his or her medical regimen.

Uniquely, CMR is well-suited to impact the detection of CV injury after receipt of cancer treatment. CMR does not incorporate ionizing radiation, thus is useful for repetitive evaluations [63]. During a single exam, both the heart and vasculature can be simultaneously assessed, an important feature when evaluating patients receiving multiple therapies that can promote injury to multiple components of the CV system, such as cardiomyocytes and arterial endothelial cells that can both experience mitochondrial dysfunction after the administration of anthracycline chemotherapy [64,65]. In addition, CMR can be utilized to detect multiple aspects of a disease process by characterizing tissue [66], measuring function [67], and identifying structural or metabolic abnormalities [58] that can be impacted by the treatment of cancer.

### CMR to detect CV related injury

**Figure 1:**
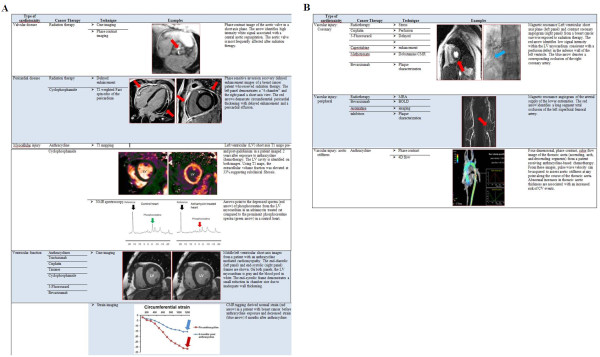
**Cardiac toxicity by type of structure affected, along with causative cancer therapies is listed below.** Valvular, pericardial and myocardial disease is shown in **A**, while vascular injury pertaining to the coronary, peripheral and aortic circulation is shown in **B**. Sample cases with a brief description of the images and the specific techniques used are shown.

The role of CMR in identifying cardiotoxicity can be divided into 5 broad categories (Figure 1A and B):

(1) The detection of cardiac anatomic or structural abnormalities including valvular lesions, pericardial disease, or evidence of metastasis;

(2) The identification and surveillance of myocellular injury;

(3) The surveillance of ventricular function;

(4) The assessment of vascular injury; and

(5) The evaluation of skeletal muscle injury.

We discuss these categories in more detail in the sections below.

#### Valvular and pericardial disease

In addition to cardiomyopathy and development of CAD, valvular and pericardial involvement has also been identified as progressive complications occurring late (i.e. 15–20 years) after exposure to chemotherapy and radiation therapy [68]. Schellong, et al. have shown that in 1,132 survivors of Hodgkin’s disease who received anthracycline and radiation before 18 years of age, valvular defects were diagnosed most frequently, followed by CAD, cardiomyopathies, conduction disorders, and pericardial abnormalities with median interval between therapy and onset of cardiac disease at 20 years. At 25 years post chemotherapy and radiation therapy, the cumulative incidence of cardiac disease was 0.14, valvular disease was 0.09 and pericardial disease was 0.05 [69]. Aortic regurgitation was the most common abnormality identified in this cohort. Using echocardiography, in 116 patients, Wethal, et al. have identified 33% progression of aortic regurgitation (defined as new onset of aortic regurgitation on follow-up, or increase by one grade of severity) and 39% development of aortic stenosis over 12 years in young survivors (median age 22 years) of Hodgkin’s lymphoma exposed to anthracyclines or radiation therapy. In a similar study of 1,249 survivors of Hodgkin’s lymphoma, absolute excess risks of requiring valve replacement or pericardiectomy/pericardiocentesis were 14.1 and 4.7 per 10,000 person-years, respectively. Extremes of age (the very old and the very young) as well as male gender were predictors for these cardiac events [70].

#### Cardiac and pericardiac metastases

**Figure 2:**
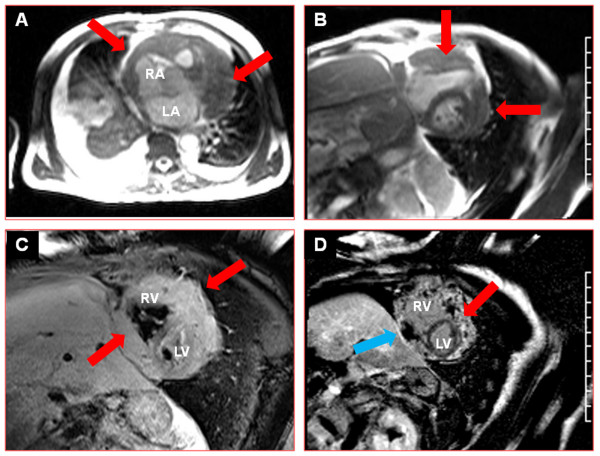
**Imaging of cardiac metastases.** Images from a patient with lung cancer with pericardial metastases. Panel **A** shows an axial slice of the heart acquired by steady-state free precession imaging. The red arrows point to the circumferential pericardial mass, which is hypointense and encases the entire right atrium, right ventricle and left ventricle. Panel **B** shows a cine image of the pericardial mass in a short-axis orientation (an additional movie file shows the cine series in motion [see Additional file 1]). In addition to the circumferential extent of the mass, the anterolateral wall of the left ventricle and the free wall of the right ventricle are tethered to the mass with reduced wall thickening and motion (arrows). Panel **C** shows a T2 weighted image in the same short-axis orientation. The red arrows point to the hyperintense mass. Panel **D** shows a delayed enhancement image of the mass in the short axis orientation. The red arrow points to the areas of hyperenhancement within the mass. The blue arrow points to the necrotic areas within the mass which are hypoenhanced with low signal intensity.

In addition to detection of valvular or pericardial anatomic abnormalities, CMR is useful in detecting tumor metastasis. While primary tumors involving the heart are relatively rare [71], metastatic involvement is not uncommon [65]. Metastases can occur through direct invasion, lymphatic or hematogenous spread, or transvenous extension [71]. Metastases to the heart and pericardium are identified at autopsy in 10%–12% of all patients with malignancies [71,72]. The most common cardiac manifestation is development of a pericardial effusion which occurs through direct invasion or lymphatic spread. Due to proximity, lung cancer is a common etiology of metastases to the heart and pericardium. In addition, breast tumors, malignant melanoma, renal cell carcinoma, mediastinal lymphomas, and leukemias can metastasize to the heart. While these are initially identified by echocardiography, CMR offers volumetric coverage of the entire heart and provides the necessary means to characterize the abnormal metastatic tissue through the use of cine imaging (see Additional file 1), T2 and T1 mapping, gadolinium enhanced perfusion, and late gadolinium enhancement as shown in Figure 2. These techniques facilitate the differentiation of a malignant tumor in comparison to a lipoma, thrombus or pericardial cyst.

#### Myocellular injury

Of all the chemotherapeutic agents, anthracyclines have been the most extensively studied in terms of myocardial histopathologic changes in the short and long-term. As shown by Billingham and Isner, et al. [52,73] some individuals do not, whereas others do, exhibit evidence of myocellular injury (myofibrillar loss, myocyte vacuolization, cellular necrosis and perivascular and interstitial fibrosis) after anthracycline exposure. With myocyte loss and interstitial edema, there is an expansion of the myocardial extracellular space and thereby the volume of distribution of water. This leads to the prolongation of T1 and T2 and thus increased signal intensity on T1 and T2 weighted images and signal prolongation (in msec) on T1 and T2 maps.

Evidence that changes in T1 signal could be appreciated with CMR were identified as early as 1987 when Thompson, et al. reported changes in pre-contrast T1 in a model of chronic adriamycin cardiotoxicity (Table 2) [74]. These investigators identified a significant prolongation of pre-contrast T1 in an ex-vivo model of chronic adriamycin toxicity in rats. In these same animals, changes in T2 were absent. The results of this study also identified abnormalities in myocardial energy metabolism using P31 nuclear magnetic resonance [58]. In this chronic adriamycin toxicity model, hemodynamic stress in the form of rapid atrial pacing was administered. Decreased myocardial phosphocreatinine levels were noted after hemodynamic stress in both the adriamycin-treated and the control rats. However, the adriamycin-treated rats experienced a delay and impartial recovery of the phosphocreatinine levels compared to controls. Thus after chronic receipt of adriamycin, both T1 tissue characteristics and cellular metabolism became abnormal after stress.

**Table 2:** Changes in Myocardial signal characteristics after cancer treatment

**Study**	**Cancer therapy**	**Myocardial signal characterization**	**How assessed**	**Subjects (# and type)**	**Findings**
Thompson et al. [74]	Anthracycline chronic toxicity	T1 changes, no T2 changes	Ex vivo spin echo	Rat model	Prolongation of pre-contrast T1.
Cottin et al. [75]	Anthracycline acute toxicity, 1 week	T1 and T2 changes	Ex vivo inversion recovery for T1, spin echo for T2	Rat model, n = 23	Prolongation of pre-contrast T1 and T2.
Wassmuth et al. [76]	Anthracycline acute toxicity, Day 3 and 28	T1 changes on Day 3	Contrast enhanced Spin echo	Humans, n = 79	Higher signal intensities on T1 weighted imaging.
Lightfoot et al. [77]	Anthracycline, acute toxicity at 2, 4, 7 and 10 weeks	T1 changes at 2 weeks and 4 weeks	Post-contrast T1 weighted inversion recovery	Rat model, n = 40	Higher signal intensity than control rats.
					This occurred early after chemotherapy and prior to a drop in the LVEF.
					This increase in signal intensity was associated with microscopic evidence of cell injury.

The observation of increased myocardial T1 on pre-contrast images was confirmed by Cottin, et al. as early as 1 week after administration of adriamycin (Table 2) [75]. This group also noted an increase in myocardial water content, and dissimilar to the Thompson results, an increase in T2, localized to the lateral free wall. These abnormalities occurred prior to deterioration in LV systolic performance. This group also identified abnormalities of lipid peroxidation concomitant with the observed increase in myocardial T1 and T2 signal.

Similarly, abnormalities in signal intensity on post-contrast T1 weighted images were noticed in human studies by Wassmuth, et al. [76]. This group of investigators used contrast enhanced spin echo in 22 patients who were imaged at 3 and 28 days after receipt of anthracycline chemotherapy. Higher myocardial signal intensities were noted as early as 3 days after receipt of anthracycline chemotherapy and predicted a future drop in the LVEF. Furthermore, patients who did not experience contrast enhancement maintained their LVEF after receipt of anthracycline chemotherapy.

**Figure 3:**
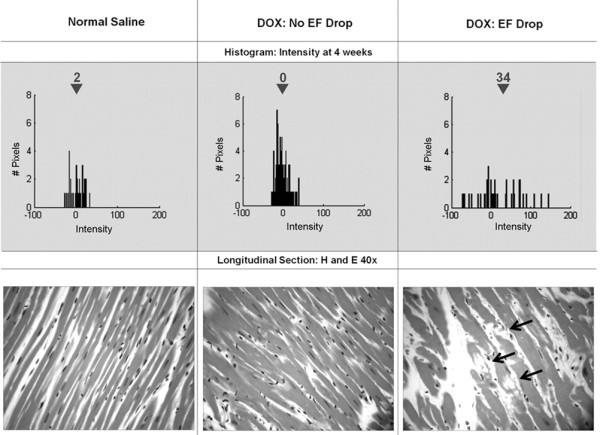
**Correlation of T1 signal intensity change after anthracycline exposure with histopathologic changes in the myocardium.** Serial histograms and histopathology of the myocardium before and after receipt of anthracycline. On the top portion of the figure are shown 4-week histograms of the number of pixels (y-axes) and intensities (x-axes) in individual animals after receipt of NS (top left), DOX without an EF drop (top middle), and DOX with an EF drop (top right). Below the histograms are 40× hematoxylin and eosin histopathologic images from the same animals. As shown, mean intensity increased in the animals that had a drop in EF corresponding to vacuolization (arrows, bottom right). *Reprinted from Lightfoot, et al. Circ Cardiovasc Imaging 2010*[77]*.*

Subsequently, with the advent of inversion recovery gradient recalled echo (GRE) sequences and the ability to identify myocardial fibrosis with gadolinium contrast, a study was performed by Lightfoot, et al. with a larger number of animals to assess the changes in myocardial T1 after varying doses of the anthracycline doxorubicin (Table 2) [77]. The changes in signal intensity in post-contrast T1 weighted images in 40 rats exposed to two different doses of doxorubicin (1.5 and 2.5 mg/kg/week) were measured and compared to that from animals that received saline. Animals that experienced a drop in the LVEF after receipt of low or high doses of doxorubicin demonstrated higher mean signal intensity measured in the post-gadolinium T1 weighted inversion recovery images of the LV myocardium relative to those animals whose LVEF was maintained (Figure 3). Furthermore, increases in signal intensity during early measurement points were 80% sensitive and 82% specific for forecasting a future drop in LVEF. As shown in Figure 3, animals with higher signal intensities were found to have typical microscopic evidence of myocellular injury due to myocardial vacuolization and the accumulation of intra- and extracellular edema. Animals without cardiac dysfunction exhibited normal signal intensities and no histopathologic evidence of myocellular injury (Figure 3).

With myocyte loss and interstitial edema, there is an expansion of the myocardial extracellular space and hence the volume of distribution of gadolinium contrast. This enables incorporation of these T1 mapping techniques for assessment of diffuse myocardial fibrosis in routine clinical studies. While conventional delayed enhancement imaging is excellent for the detection of focal myocardial fibrosis, this requires nulling of the remaining myocardium rendering it more difficult to assess diffuse myocardial fibrosis. Multiple studies have confirmed the utility of T1 mapping before and after gadolinium to identify the myocardial partition coefficient of gadolinium and thereby the myocardial extracellular volume fraction (ECVF) [78-80]. Messroghli, et al. have developed accurate T1 mapping techniques to be performed in a single breathhold [81-83], and Flett, et al. have studied alternative T1 mapping techniques and found an excellent correlation between diffuse fibrosis and the histological collagen volume fraction in patients with myocardial hypertrophy due to aortic stenosis or hypertrophic cardiomyopathy [84]. In patients with cardiomyopathy, the myocardial ECVF assessment has prognostic value beyond assessment of LVEF [85]. Whether similar prognostic value of the myocardial ECVF can be assessed in cancer survivors is an area of ongoing study.

In summary, CMR has the potential to assess early cardiac injury by using T2 mapping and pre- and post-contrast T1 mapping. This ability to identify myocardial abnormalities that precede functional impairment offers an opportunity to study the benefit of starting cardioprotective agents before overt functional deterioration.

#### Ventricular function

With the realization that evidence of myocardial injury due to the administration of anthracyclines could be appreciated during invasive endomyocardial biopsies or from diagnostic catheterization procedures, investigators sought to develop non-invasive methodologies to accomplish early detection of chemotherapy-related myocardial injury. In 1979, Alexander, et al. utilized quantitative, multi-gated radionuclide angiocardiography (or MUGA) scanning to serially assess LVEF in patients scheduled to receive doxorubicin for treatment of cancer. In a cohort of 55 individuals, a decline in LVEF by at least 15% to a final value of <45% was associated with the development of CHF in patients receiving >350 mg/m^2^ of anthracycline chemotherapy [53,86]. Soon thereafter, other investigators confirmed these findings [57].

As a result of these and other early non-invasive imaging studies, guidelines were published in 1992 that highlighted the use of LVEF measurements to screen and monitor patients for the development of cardiotoxicity and chemotherapy associated CHF [57,86]. These guidelines included an assessment of baseline LVEF prior to chemotherapy followed by subsequent LVEF measurements depending on the frequency and cumulative anthracycline dose prescribed. During serial surveillance, if a patient developed a deterioration in LVEF, then the risk of developing CHF was elevated and therapeutic interventions were suggested. It was also recognized that the surveillance strategy could be modified due to the presence of potential additional risk factors for anthracycline-based myocardial injury. These risk factors included the application of mediastinal radiation, age (the very young and those with advanced age), pre-existing heart disease, and the associated administration of cyclophosphamide. Today, the majority of these early established guidelines are pervasive in the application of clinical care for patients that will receive anthracycline-based chemotherapy for the treatment of cancer.

Recent trials of newer cancer therapies that also promote cardiac injury such as trastuzumab, also routinely monitor LVEF with echocardiography or MUGA scanning to detect early deteriorations in LVEF that predispose one to develop CHF [36]. Guidelines have been published for the surveillance of LVEF during trastuzumab or other cancer therapies by the UK National Cancer Institute [87], and by the European Society of Medical Oncology [88]. In addition to baseline assessments of LVEF, additional assessments are recommended at 3, 6, and 9 months after treatment with anthracyclines, and at 4 monthly intervals for trastuzumab. Furthermore, the UK National Cancer Institute guidelines detail the use of a “traffic light” system to enable detection and management of cardiotoxicity with trastuzumab in which the therapeutic administration of angiotensin converting enzyme inhibitors is directed through measurement of LVEF.

CMR is well-suited to assess LVEF prior to and during receipt of potentially cardiotoxic chemotherapy. The ability to acquire images in multiple tomographic planes without limitations imposed by body habitus enable CMR LVEF assessments to be performed in patients who may have undergone prior surgery to pericardiac regions including the chest, lungs and mediastinum. Importantly, CMR accurately measures the ventricular volumes that contribute to the LVEF calculation. Compared with two-dimensional (2D) echocardiography, the inter-study coefficient of variability for assessment of LV volumes, function and mass was superior for CMR among healthy individuals, as well as those with heart failure or left ventricular hypertrophy [89]. The inter-study reproducibility for CMR measured end-systolic volume was 4.4% to 9.2% (versus 12.7 to 20.3% for 2D echocardiography), for ejection fraction was 2.4% to 7.3% (versus 8.6% to 9.4% for 2D echocardiography), and for LV mass was 2.8% to 4.8% (versus 11.6% to 15.7% for 2D echocardiography) [89]. Similarly, low inter-observer variability was noted in a comprehensive study comparing CMR volumes derived by cine and spin echo CMR [90]. Abnormalities of left ventricular end-diastolic volume (LVEDV), which could be due to changes in LV pre-load, can be detected with CMR. This LVEDV assessment is particularly important in patients receiving treatment for cancer who may exhibit altered pre-load due to poor oral intake or excessive nausea and vomiting. In addition, CMR can accurately identify abnormalities of left ventricular end-systolic volume (LVESV), which reflect abnormalities of myocardial contractility. Finally, the 3-dimensional acquisitions of these LV volumes and EF are very useful when cardiac function becomes reduced and the left ventricle assumes an abnormal shape that may differ from a prolate ellipsoid that is often assumed for the calculation of LVEF using a 2D technique.

**Figure 4:**
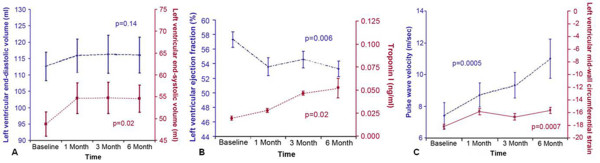
**Serial changes in LV volumes, myocardial strain and LVEF after anthracycline exposure in human subjects.** Time dependent changes in left ventricular (LV) end diastolic volume (left y-axis; panel **A**) and LV end systolic volume (right y-axis; panel **A**); in LV ejection fraction (left y-axis; panel **B**); serum Troponin-I (right y-axis; panel **B**); pulse wave velocity (PWV) (left y-axis; panel **C**); and mean mid wall circumferential strain (right y-axis; panel **C**). The mean ± the standard error are shown. There was a substantive increase in LV end systolic volume, PWV, and serum Troponin-I while at the same time a decrease in LV ejection fraction and mean mid-wall circumferential strain from baseline to six months after administration of low to moderate doses of anthracycline-based chemotherapy. *Reprinted from Drafts, et al. JACC-Imaging 2012*[96]*.*

In addition to determinations of LV volumes and EF, other measures of LV systolic and diastolic function can be obtained during the same CMR examination. For example, myocardial strain, a unitless measure of myocardial deformation can be assessed during ventricular systole or during ventricular diastole (strain rate). Both of these measures may provide incremental diagnostic information relative to LV function [91]. These values have been useful in assessing and identifying individuals with abnormalities of LV function due to ischemic [92] and non-ischemic cardiomyopathies [93] as well as other infiltrative myocardial diseases such as systemic amyloidosis [94,95]. Recently, Drafts, et al. acquired serial assessments of LV volumes and myocardial strain to identify abnormalities of LV performance during receipt of anthracycline chemotherapy. In 55 individuals scheduled to receive an anthracycline-based chemotherapeutic regimen for leukemia, lymphoma, or breast cancer, Drafts, et al., found that measures of LV systolic performance (LVESV and myocardial strain) deteriorated early (even after the first dose) of the administration of anthracycline-based chemotherapy (Figure 4) [96]. Moreover, these deteriorations remained present after the discontinuation of anthracycline chemotherapy. These data highlight the potential utility of CMR for identifying abnormalities of LV performance that indicate ongoing cardiac dysfunction due to toxicity from anthracycline-based regimens. Combining both these advanced functional measures with myocardial tissue characterization techniques (such as the mapping technologies mentioned previously) could provide a new strategy for identifying those at risk of CHF and thereby guide therapeutic interventions to reduce these risks. Currently, a large multi-center randomized trial is underway utilizing CMR to assess the efficacy of conventional heart failure drugs such as ACEIs and beta-blockers in patients receiving trastuzumab therapy for breast cancer [97].

In addition to myocardial strain and volume assessment, promising techniques being developed include diffusion tensor imaging for the identification of impaired myocardial mechanics, [98] for example, impaired torsion and spectroscopy for assessing abnormal myocardial metabolism [99]. These techniques are currently being studied in hypertrophic cardiomyopathy and could be potentially used in patients exposed to cancer therapies, to identify myocardial abnormalities prior to the onset of overt systolic dysfunction.

#### Vascular injury

It is important to recognize that in addition to the development of CHF, many treatments for cancer also predispose individuals to the development of CV events related to abnormalities of the vascular system. These CV events include the development of MI, PAD, and stroke. Several different cancer therapies have been shown to promote these vascular related events.

**Figure 5:**
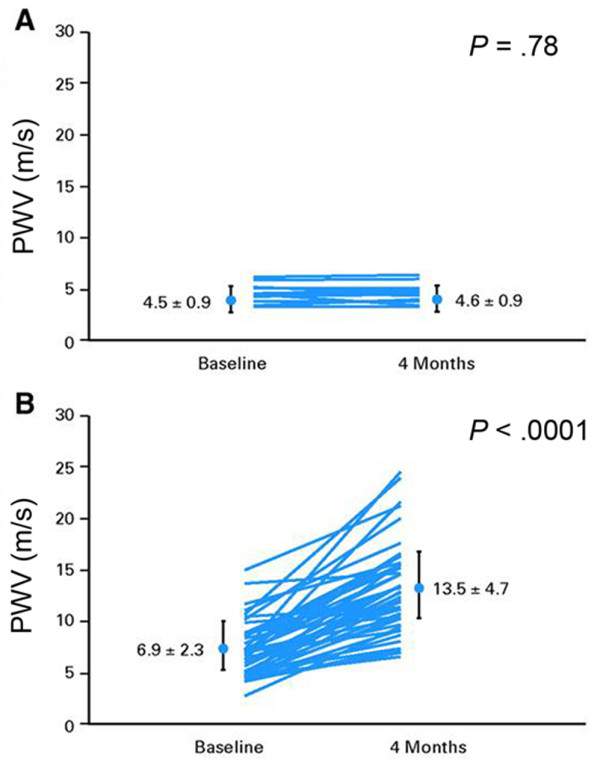
**Serial changes in aortic stiffness in chemotherapy recipients.** Chaosuwannakit, et al. demonstrated that pulse wave velocity (PWV) increased in participants receiving cancer therapy. PWV results for the control participants **(A)** and the participants receiving cancer therapy **(B)**. The increase in PWV observed in this study is associated with a 3-fold increase in the risk of a future CV event and consistent with a 10 to 20 year age associated increment of the cardiovascular system. *Reprinted from Chaosuwannakit, et al. J Clin Oncol 2010*[64]*.*

Hormonal therapy such as ADT is associated with an increased incidence of MI, PAD, and stroke [45,46,100]. Tyrosine kinase inhibitors such as bevacizumab, sorafinib and sunitinib promote hypertension [100-103]. Recently, Chaosuwannakit, et al. demonstrated that proximal aortic wall stiffness increased 3 months after receipt of anthracycline-based chemotherapy compared to age matched controls (Figure 5) [64]. Among the anthracycline-based chemotherapy recipients, the increased aortic stiffness was noted even after controlling for factors such as age, gender, diabetes, hyperlipidemia and hypertension. The magnitude of the increase was equivalent to that associated with aging the CV system by 10–20 years.

Two additional features of this increase in stiffness are noteworthy. First, this occurred soon after administration of chemotherapy and therefore is dissimilar to the more chronic causes of stiffening (e.g. atherosclerosis) more commonly observed. Recently, Eckman, et al. identified early histopathologic evidence of coronary artery microcirculatory endothelial damage in an animal model of anthracycline cardiotoxicity [104]. Perhaps endothelial damage to the vasa vasorum supply or on the luminal surface of the aorta contributed to the abrupt increase in aortic stiffening. Further research is needed in this area.

Second, the increases in aortic stiffening were not dose dependent. This finding suggests there may be thresholds of susceptibility to vascular dysfunction, and further research may be useful in identifying these susceptibilities. These increases in aortic stiffness could have important clinical ramifications. Abnormal increases in proximal aortic stiffness have been associated with LV hypertrophy, exercise capacity (particular in the elderly) [105], and future CV events in those with diabetes, hypertension, renal failure, and advanced age. It is important to note, however, that it is unknown whether these increases in aortic stiffness are transient or whether they reverse after changes in therapy.

Additional changes to the vascular system have been reported upon receipt of treatment for cancer. These include the development of accelerated atherosclerosis and abnormalities of peripheral arterial endothelial function. In men treated for prostate cancer and post-menopausal women treated for breast cancer, the administration of ADT men and estrogen deprivation for women have been associated with the development of metabolic syndrome, diabetes, atherosclerosis, and CV events. Although not studied previously, there is potential for CMR to be used to identify the rapid progression of atherosclerosis by identifying abnormalities of aortic or carotid arterial wall thickness or the components of plaque composition (Figure 1B).

The administration of anthracycline-based chemotherapy has been associated with abnormalities of peripheral arterial and endothelial function. Endothelial function can be assessed noninvasively through the assessment of flow mediated arterial dilation (FMAD). CMR is highly advantageous for the assessment of peripheral arterial FMAD (Figure 1B). Phase contrast techniques that measure both wall shear stress and arterial dilation can be performed prior to, during, and after cuff inflation on both the upper as well as the lower extremity. These measures have been useful in identifying abnormalities of FMAD in older individuals with heart failure due to a reduced or preserved LVEF. At present, CMR FMAD measurements have not been acquired in patients treated for cancer. As the number of therapies targeted toward the vascular supply of tumors increases, the proven utility of CMR for assessing peripheral arterial endothelial function may be useful for identifying unintended injury to the native arterial circulation.

#### Skeletal muscle function as a contributor to exercise capacity and fatigue

Fatigue and muscle weakness are common among recipients of chemotherapy, and often they persist for many years after cessation of cancer treatment [106-108]. Perceived fatigue and weakness is debilitating with a profoundly negative impact on quality of life. In 33% of women surviving treatment for breast cancer, fatigue is progressive and severely limits the ability to return to work [106]. In addition, fatigue may influence exercise capacity, a known predictor of CV events. Patients who received chemotherapy experience both fatigue and exercise intolerance, and therapeutic interventions that modify exercise capacity in cancer survivors also improve fatigue [109]. In fact, physical activity >3 METS is associated with reduced CV related morbidity and mortality in survivors of both breast and colon cancer [110,111].

In those capable of normal ambulation, exercise capacity is influenced during stress by changes in cardiac output, arterial and microcirculatory function, and skeletal muscle metabolism and function. CMR is well-suited to assess all three of these components of exercise capacity both at rest and during pharmacologic or exercise induced stress. In elderly patients with heart failure and preserved or reduced EF [112,113], CMR derived information from these three components has been utilized to understand mechanisms of disability and exercise intolerance.

In addition to the cancer therapy mediated cardiac and vascular abnormalities identified in Table 1, skeletal muscle dysfunction may also occur after treatment for cancer. For example, doxorubicin potentiates direct skeletal muscle weakness through generation of reactive oxygen species and TNFα signaling [114]. Anthracyclines accumulate in skeletal muscle and have been shown to decrease muscle force on direct muscle force testing [115]. In addition they also cause loss of myofibrillar organization and interstitial edema [116]. To date, the role of the skeletal muscle weakness in exercise intolerance in chemotherapy recipients has not been systematically addressed; however, skeletal muscle energetics assessed at rest or after exercise with multi-nuclear spectroscopy could be highly informative in detecting abnormalities of ATP utilization that occur as a result of the impact of oxidative stress on the working mitochondria (Figure 1A). In addition, skeletal muscle oxygen uptake can be studied with novel blood oxygen level dependent (BOLD) MRI techniques. BOLD signaling relies on the hemoglobin oxygen saturation [117]. During increased tissue perfusion at constant levels of oxygen extraction, the oxyhemoglobin concentration is higher, and a lower concentration of paramagnetic deoxyhemoglobin is noted. This results in an increase in the T2 and T2* signal (positive BOLD signal). Coupling these BOLD assessments with measurements of skeletal muscle level microcirculatory perfusion after exercise using arterial spin labeling would be helpful to delineate muscle oxygen extraction for given levels of perfusion. In the calf of individuals with PAD, skeletal muscle phosphocreatine recovery after exercise has been shown to be diminished relative to control populations [118], and in combination with the measures of perfusion, oxygen utilization, and skeletal muscle mass, the incorporation of energetics could provide a more complete and quite novel understanding of the etiology of fatigue in cancer survivors (Figure 1A and B).

Future directions:

We have identified areas of research in cardiovascular toxicity beyond the assessment of cardiac structure and function, specifically to explore the mechanisms leading to co-morbidities that have a delayed onset after the initial exposure to cancer therapeutics. Table 3 provides a list of available newer MRI tools that can be utilized to assess the etiology of fatigue, accelerated atherosclerosis of the coronary, cerebral and peripheral vascular systems in addition to accelerated stiffness of the entire vascular system.

**Table 3:** Unresolved issues in cancer survivors and possible techniques to address these issues

**Morbidity**	**Unresolved question**	**Proposed MRI technique**
Cerebro vascular accident	Accelerated atherosclerosis	Wall thickness of ascending, descending aorta, plaque characterization
Myocardial infarction		Coronary flow reserve using quantitative myocardial perfusion
Peripheral vascular disease		Wall thickness of femoral arteries, BOLD imaging
LV systolic dysfunction	Early detection of myocellular injury and risk stratification	T1, T2 and ECV mapping
		Strain imaging
	Impairment of myocardial mechanics, i.e. Impaired torsion	Diffusion tensor imaging
	Myocardial energy metabolism	NMR P32 spectroscopy
Impaired exercise tolerance	Arterial stiffness	4D Flow for assessing pulse wave velocity
Fatigue	Skeletal muscle injury	NMR spectroscopy to assess for mitochondrial dysfunction in skeletal muscle
		Fat/water separation to assess fat content
Hypertension	Endothelial dysfunction	Flow mediated arterial dilation

## Conclusion

In summary, although cancer free survival is improving for many individuals treated for malignancies, an unintended consequence has been the emergence of cardiovascular events in the form of heart failure, myocardial infarction, stroke, and peripheral arterial disease. Temporally, it appears that these cardiovascular events and underlying subclinical cardiovascular disease are related to the therapies received for cancer. To this end, the ability of cardiovascular magnetic resonance to characterize and assess the function of the cardiovascular system is useful in identifying subclinical abnormalities of cardiovascular function that often precede CV events. Several single center studies have demonstrated the utility of CMR technologies to identify this early subclinical cardiovascular injury. Importantly however, further research is needed to fully develop cardiovascular magnetic procedures that both diagnose and also guide therapeutic interventions to prevent cardiovascular event and also the pronounced fatigue and related morbidities associated with administration of therapy for cancer.

## Competing interests

Both authors declare thats they have no competing interests.

## Authors’ contributions

Both authors conceived the manuscript, performed the background research and review, and wrote and edited the text. Both authors read and approved the final manuscript.

## Supplementary Material

Additional file 1 **Pericardial mass cineseries.** Advanced Systems Format File (.asf) showing cine image of the pericardial mass in a short-axis orientation. Click here for file

## References

[B1] Bowles EJ Wellman R Feigelson HS Onitilo AA Freedman AN Delate T Allen LA Nekhlyudov L Goddard KA Davis RL Risk of heart failure in breast cancer patients after anthracycline and trastuzumab treatment: a retrospective cohort study J Natl Cancer Inst 2012 104 1293 305 10.1093/jnci/djs317 22949432 PMC3433392

[B2] Brenner H Long-term survival rates of cancer patients achieved by the end of the 20th century: a period analysis Lancet 2002 360 1131 5 10.1016/S0140-6736(02)11199-8 12387961

[B3] Coughlin SS Ekwueme DU Breast cancer as a global health concern Cancer Epidemiol 2009 33 315 8 10.1016/j.canep.2009.10.003 19896917

[B4] Du XL Fox EE Lai D Competing causes of death for women with breast cancer and change over time from 1975 to 2003 Am J Clin Oncol 2008 31 105 16 10.1097/COC.0b013e318142c865 18391593 PMC2570158

[B5] Geiger AM Trastuzumab and congestive heart failure: what can we learn from use in the community? J Natl Cancer Inst 2012 104 1269 70 22949431 10.1093/jnci/djs342 PMC3840575

[B6] Hooning MJ Botma A Aleman BM Baaijens MH Bartelink H Klijn JG Taylor CW van Leeuwen FE Long-term risk of cardiovascular disease in 10-year survivors of breast cancer J Natl Cancer Inst 2007 99 365 75 10.1093/jnci/djk064 17341728

[B7] Maurea N Coppola C Ragone G Frasci G Bonelli A Romano C Iaffaioli RV Women survive breast cancer but fall victim to heart failure: the shadows and lights of targeted therapy J Cardiovasc Med (Hagerstown) 2010 11 861 8 10.2459/JCM.0b013e328336b4c1 20072001

[B8] Patnaik JL Byers T DiGuiseppi C Dabelea D Denberg TD Cardiovascular disease competes with breast cancer as the leading cause of death for older females diagnosed with breast cancer: a retrospective cohort study Breast Cancer Res 2011 13 R64 10.1186/bcr2901 21689398 PMC3218953

[B9] Pinder MC Duan Z Goodwin JS Hortobagyi GN Giordano SH Congestive heart failure in older women treated with adjuvant anthracycline chemotherapy for breast cancer J Clin Oncol 2007 25 3808 15 10.1200/JCO.2006.10.4976 17664460

[B10] Schultz PN Beck ML Stava C Vassilopoulou-Sellin R Health profiles in 5836 long-term cancer survivors Int J Cancer 2003 104 488 95 10.1002/ijc.10981 12584748

[B11] Sukel MP Breekveldt-Postma NS Erkens JA van der Linden PD Beiderbeck AB Coebergh JW Herings RM Incidence of cardiovascular events in breast cancer patients receiving chemotherapy in clinical practice Pharmacoepidemiol Drug Saf 2008 17 125 34 10.1002/pds.1528 18058835

[B12] Yood MU Wells KE Alford SH Dakki H Beiderbeck AB Hurria A Gross CP Oliveria SA Cardiovascular outcomes in women with advanced breast cancer exposed to chemotherapy Pharmacoepidemiol Drug Saf 2012 21 818 27 10.1002/pds.3239 22419528 PMC3785307

[B13] SEER Cancer Statistics Review, 1975–2008 http://seer.cancer.gov/csr/1975_2008/, based on November 2010 SEER data submission, posted to the SEER web site, 201118718969

[B14] Cancer Facts & Figures 2012 http://www.cancer.org/acs/groups/content/@epidemiologysurveilance/documents/document/acspc-031941.pdf23910930

[B15] Smith LA Cornelius VR Plummer CJ Levitt G Verrill M Canney P Jones A Cardiotoxicity of anthracycline agents for the treatment of cancer: systematic review and meta-analysis of randomised controlled trials BMC Cancer 2010 10 337 10.1186/1471-2407-10-337 20587042 PMC2907344

[B16] Seidman A Hudis C Pierri MK Shak S Paton V Ashby M Murphy M Stewart SJ Keefe D Cardiac dysfunction in the trastuzumab clinical trials experience J Clin Oncol 2002 20 1215 21 10.1200/JCO.20.5.1215 11870163

[B17] Keating NL O’Malley AJ Smith MR Diabetes and cardiovascular disease during androgen deprivation therapy for prostate cancer J Clin Oncol 2006 24 27 4448 56 10.1200/JCO.2006.06.2497 16983113

[B18] Pugh TJ Ballonoff A Rusthoven KE McCammon R Kavanagh B Newman F Rabinovitch R Cardiac mortality in patients with stage I and II diffuse large B-cell lymphoma treated with and without radiation: a surveillance, epidemiology, and end-results analysis Int J Radiat Oncol Biol Phys 2010 76 845 9 10.1016/j.ijrobp.2009.02.045 19515509

[B19] Darby SC Ewertz M McGale P Bennet AM Blom-Goldman U Brønnum D Correa C Cutter D Gagliardi G Gigante B Risk of ischemic heart disease in women after radiotherapy for breast cancer N Engl J Med 2013 368 987 998 10.1056/NEJMoa1209825 23484825

[B20] Meacham LR Chow EJ Ness KK Kamdar KY Chen Y Yasui Y Oeffinger KC Sklar CA Robison LL Mertens AC Cardiovascular risk factors in adult survivors of pediatric cancer–a report from the childhood cancer survivor study Cancer Epidemiol Biomarkers Prev 2010 19 170 81 10.1158/1055-9965.EPI-09-0555 20056636 PMC2805162

[B21] Doyle JJ Neugut AI Jacobson JS Grann VR Hershman DL Chemotherapy and cardiotoxicity in older breast cancer patients: a population-based study J Clin Oncol 2005 23 8597 605 10.1200/JCO.2005.02.5841 16314622

[B22] Diller L Chow EJ Gurney JG Hudson MM Kadin-Lottick NS Kawashima TI Leisenring WM Meacham LR Mertens AC Mulrooney DA Chronic disease in the childhood cancer survivor study cohort: a review of published findings J Clin Oncol 2009 27 2339 55 10.1200/JCO.2008.21.1953 19364955 PMC2677922

[B23] Guarneri V Lenihan DJ Valero V Durand JB Broglio K Hess KR Michaud LB Gonzalez-Angulo AM Hortobagyi GN Esteva FJ Long-term cardiac tolerability of trastuzumab in metastatic breast cancer: the M.D. Anderson Cancer Center experience J Clin Oncol 2006 24 4107 15 10.1200/JCO.2005.04.9551 16908934

[B24] Mulrooney DA Yeazel MW Kawashima T Mertens AC Mitby P Stovall M Donaldson SS Green DM Sklar CA Robison LL Leisenring WM Cardiac outcomes in a cohort of adult survivors of childhood and adolescent cancer: retrospective analysis of the Childhood Cancer Survivor Study cohort BMJ 2009 339 b4606 10.1136/bmj.b4606 19996459 PMC3266843

[B25] Perez EA Rodeheffer R Clinical cardiac tolerability of trastuzumab J Clin Oncol 2004 22 322 9 14722042 10.1200/JCO.2004.01.120

[B26] Steinberger J Sinaiko AR Kelly AS Leisenring WM Steffen LM Goodman P Mulrooney DA Dietz AC Moran A Perkins JL Baker KS Cardiovascular risk and insulin resistance in childhood cancer survivors J Pediatr 2011 160 494 9 21920542 10.1016/j.jpeds.2011.08.018 PMC3246569

[B27] Swerdlow AJ Higgins CD Smith P Cunningham D Hancock BW Horwich A Hoskin PJ Lister A Radford JA Rohatiner AZ Linch DC Myocardial infarction mortality risk after treatment for Hodgkin disease: a collaborative British cohort study J Natl Cancer Inst 2007 99 206 14 10.1093/jnci/djk029 17284715

[B28] Ng AK Bernardo MP Weller E Backstrand KH Silver B Marcus KC Tarbell NJ Friedberg J Canellos GP Mauch PM Long-term survival and competing causes of death in patients with early-stage Hodgkin’s disease treated at age 50 or younger J Clin Oncol 2002 20 2101 8 10.1200/JCO.2002.08.021 11956271

[B29] Bouchardy C Rapiti E Usel M Majno SB Vlastos G Benhamou S Miralbell R Neyroud-Caspar I Verkooijen HM Vinh-Hung V Excess of cardiovascular mortality among node-negative breast cancer patients irradiated for inner-quadrant tumors Ann Oncol 2010 21 459 65 10.1093/annonc/mdp341 19703922

[B30] Bouillon K Haddy N Delaloge S Garbay JR Garsi JP Brindel P Mousannif A Lê MG Labbe M Arriagada R Long-term cardiovascular mortality after radiotherapy for breast cancer J Am Coll Cardiol 2011 57 445 52 10.1016/j.jacc.2010.08.638 21251585

[B31] Curigliano G Mayer EL Burstein HJ Winer EP Goldhirsch A Cardiac toxicity from systemic cancer therapy: a comprehensive review Prog Cardiovasc Dis 2010 53 94 104 10.1016/j.pcad.2010.05.006 20728696

[B32] Roychoudhuri R Robinson D Putcha V Cuzick J Darby S Møller H Increased cardiovascular mortality more than fifteen years after radiotherapy for breast cancer: a population-based study BMC Cancer 2007 7 9 10.1186/1471-2407-7-9 17224064 PMC1784099

[B33] Yeh ET Bickford CL Cardiovascular complications of cancer therapy: incidence, pathogenesis, diagnosis, and management J Am Coll Cardiol 2009 53 2231 47 10.1016/j.jacc.2009.02.050 19520246

[B34] Crone SA Zhao YY Fan L Gu Y Minamisawa S Liu Y Peterson KL Chen J Kahn R Condorelli G ErbB2 is essential in the prevention of dilated cardiomyopathy Nat Med 2002 8 459 65 10.1038/nm0502-459 11984589

[B35] Slamon DJ Leyland-Jones B Shak S Fuchs H Paton V Bajamonde A Fleming T Eiermann W Wolter J Pegram M Use of chemotherapy plus a monoclonal antibody against HER2 for metastatic breast cancer that overexpresses HER2 N Engl J Med 2001 344 783 92 10.1056/NEJM200103153441101 11248153

[B36] Slamon D Eiermann W Robert N Pienkowski T Martin M Press M Mackey J Glaspy J Chan A Pawlicki M Adjuvant trastuzumab in HER2-positive breast cancer N Engl J Med 2011 365 1273 83 10.1056/NEJMoa0910383 21991949 PMC3268553

[B37] Piccart-Gebhart MJ Procter M Leyland-Jones B Goldhirsch A Untch M Smith I Gianni L Baselga J Bell R Jackisch C Trastuzumab after adjuvant chemotherapy in HER2-positive breast cancer N Engl J Med 2005 353 1659 72 10.1056/NEJMoa052306 16236737

[B38] Tan-Chiu E Yothers G Romond E Geyer CE Jr Ewer M Keefe D Shannon RP Swain SM Brown A Fehrenbacher L Assessment of cardiac dysfunction in a randomized trial comparing doxorubicin and cyclophosphamide followed by paclitaxel, with or without trastuzumab as adjuvant therapy in node-positive, human epidermal growth factor receptor 2-overexpressing breast cancer: NSABP B-31 J Clin Oncol 2005 23 7811 9 10.1200/JCO.2005.02.4091 16258083

[B39] Amir E Seruga B Niraula S Carlsson L Ocaña A Toxicity of adjuvant endocrine therapy in postmenopausal breast cancer patients: a systematic review and meta-analysis J Natl Cancer Inst 2011 103 1299 309 10.1093/jnci/djr242 21743022

[B40] Goss PE Ingle JN Martino S Robert NJ Muss HB Piccart MJ Castiglione M Tu D Shepherd LE Pritchard KI A randomized trial of letrozole in postmenopausal women after five years of tamoxifen therapy for early-stage breast cancer N Engl J Med 2003 349 1793 802 10.1056/NEJMoa032312 14551341

[B41] Huggins C Hodges CV Studies on prostatic cancer: I. The effect of castration, of estrogen and of androgen injection on serum phosphatases in metastatic carcinoma of the prostate. 1941 J Urol 2002 168 9 12 10.1016/S0022-5347(05)64820-3 12050481

[B42] Thurlimann B Keshaviah A Coates AS Mouridsen H Mauriac L Forbes JF Paridaens R Castiglione-Gertsch M Gelber RD Rabaglio M A comparison of letrozole and tamoxifen in postmenopausal women with early breast cancer N Engl J Med 2005 353 2747 57 16382061 10.1056/NEJMoa052258

[B43] Chapman JA Meng D Shepherd L Parulekar W Ingle JN Muss HB Palmer M Yu C Goss PE Competing causes of death from a randomized trial of extended adjuvant endocrine therapy for breast cancer J Natl Cancer Inst 2008 100 252 60 10.1093/jnci/djn014 18270335 PMC2745611

[B44] Ewer MS Gluck S A woman’s heart: the impact of adjuvant endocrine therapy on cardiovascular health Cancer 2009 115 1813 26 10.1002/cncr.24219 19235248

[B45] Hu JC Williams SB O’Malley AJ Smith MR Nguyen PL Keating NL Androgen-deprivation therapy for nonmetastatic prostate cancer is associated with an increased risk of peripheral arterial disease and venous thromboembolism Eur Urol 2012 10.1016/j.eururo.2012.01.045 PMC3719131 22336376

[B46] Keating NL O’Malley AJ Freedland SJ Smith MR Diabetes and cardiovascular disease during androgen deprivation therapy: observational study of veterans with prostate cancer J Natl Cancer Inst 2010 102 39 46 10.1093/jnci/djp404 19996060 PMC3107568

[B47] Van Hemelrijck M Garmo H Holmberg L Ingelsson E Bratt O Bill-Axelson A Lambe M Stattin P Adolfsson J Absolute and relative risk of cardiovascular disease in men with prostate cancer: results from the Population-Based PCBaSe Sweden J Clin Oncol 2010 28 3448 56 10.1200/JCO.2010.29.1567 20567006

[B48] Azoulay L Yin H Benayoun S Renoux C Boivin JF Suissa S Androgen-deprivation therapy and the risk of stroke in patients with prostate cancer Eur Urol 2011 60 1244 50 10.1016/j.eururo.2011.08.041 21908097

[B49] Buzdar A Howell A Cuzick J Wale C Distler W Hoctin-Boes G Houghton J Locker GY Nabholtz JM Comprehensive side-effect profile of anastrozole and tamoxifen as adjuvant treatment for early-stage breast cancer: long-term safety analysis of the ATAC trial Lancet Oncol 2006 7 633 43 16887480 10.1016/S1470-2045(06)70767-7

[B50] Meinardi MT van der Graaf WT van Veldhuisen DJ Gietema JA de Vries EG Sleijfer DT Detection of anthracycline-induced cardiotoxicity Cancer Treat Rev 1999 25 237 47 10.1053/ctrv.1999.0128 10448132

[B51] Billingham ME Mason JW Bristow MR Daniels JR Anthracycline cardiomyopathy monitored by morphologic changes Cancer Treat Rep 1978 62 865 72 667860

[B52] Isner JM Ferrans VJ Cohen SR Witkind BG Virmani R Gottdiener JS Beck JR Roberts WC Clinical and morphologic cardiac findings after anthracycline chemotherapy. Analysis of 64 patients studied at necropsy Am J Cardiol 1983 51 1167 74 10.1016/0002-9149(83)90364-8 6573121

[B53] Alexander J Dainiak N Berger HJ Goldman L Johnstone D Reduto L Duffy T Schwartz P Gottschalk A Zaret BL Serial assessment of doxorubicin cardiotoxicity with quantitative radionuclide angiocardiography N Engl J Med 1979 300 278 83 10.1056/NEJM197902083000603 759880

[B54] McKillop JH Bristow MR Goris ML Billingham ME Bockemuehl K Sensitivity and specificity of radionuclide ejection fractions in doxorubicin cardiotoxicity Am Heart J 1983 106 1048 56 10.1016/0002-8703(83)90651-8 6637763

[B55] Gottdiener JS Mathisen DJ Borer JS Bonow RO Myers CE Barr LH Schwartz DE Bacharach SL Green MV Rosenberg SA Doxorubicin cardiotoxicity: assessment of late left ventricular dysfunction by radionuclide cineangiography Ann Intern Med 1981 94 430 5 10.7326/0003-4819-94-4-430 7212498

[B56] Shankar SM Marina N Hudson MM Hodgson DC Adams MJ Landier W Bhatia S Meeske K Chen MH Kinahan KE Monitoring for cardiovascular disease in survivors of childhood cancer: report from the cardiovascular disease task force of the children’s oncology group Pediatrics 2008 121 e387 96 10.1542/peds.2007-0575 18187811

[B57] Steinherz LJ Graham T Hurwitz R Sondheimer HM Schwartz RG Shaffer EM Sandor G Benson L Williams R Guidelines for cardiac monitoring of children during and after anthracycline therapy: report of the cardiology committee of the childrens cancer study group Pediatrics 1992 89 942 9 1579408

[B58] Bittner V Reeves RC Digerness SB Caulfield JB Pohost GM 31P NMR spectroscopy in chronic adriamycin cardiotoxicity Magn Reson Med 1991 17 69 81 10.1002/mrm.1910170112 2067408

[B59] Cardinale D Lamantia G Cipolla CM Troponin I and cardiovascular risk stratification in patients with testicular cancer J Clin Oncol 2006 24 3508 9 16849772 10.1200/JCO.2006.06.7876

[B60] Cardinale D Salvatici M Sandri MT Review: role of biomarkers in cardioncology Clin Chem Lab Med 2011 49 1937 48 21892906 10.1515/CCLM.2011.692

[B61] Cardinale D Colombo A Cipolla CM Prevention and treatment of cardiomyopathy and heart failure in patients receiving cancer chemotherapy Curr Treat Options Cardiovasc Med 2008 10 486 95 10.1007/s11936-008-0041-x 19026179

[B62] Cardinale D Colombo A Sandri MT Lamantia G Colombo N Civelli M Martinelli G Veglia F Fiorentini C Cipolla CM Prevention of high-dose chemotherapy-induced cardiotoxicity in high-risk patients by angiotensin-converting enzyme inhibition Circulation 2006 114 2474 81 10.1161/CIRCULATIONAHA.106.635144 17101852

[B63] Hendel RC Patel MR Kramer CM Poon M Carr JC Gerstad NA Gillam LD Hodgson JM Kim RJ Lesser JR ACCF/ACR/SCCT/SCMR/ASNC/NASCI/SCAI/SIR 2006 appropriateness criteria for cardiac computed tomography and cardiac magnetic resonance imaging: a report of the American College of Cardiology Foundation Quality Strategic Directions Committee Appropriateness Criteria Working Group, American College of Radiology, Society of Cardiovascular Computed Tomography, Society for Cardiovascular Magnetic Resonance, American Society of Nuclear Cardiology, North American Society for Cardiac Imaging, Society for Cardiovascular Angiography and Interventions, and Society of Interventional Radiology J Am Coll Cardiol 2006 48 1475 97 10.1016/j.jacc.2006.07.003 17010819

[B64] Chaosuwannakit N D’Agostino R Jr Hamilton CA Lane KS Ntim WO Lawrence J Melin SA Ellis LR Torti FM Little WC Hundley WG Aortic stiffness increases upon receipt of anthracycline chemotherapy J Clin Oncol 2010 28 166 72 10.1200/JCO.2009.23.8527 19901105 PMC2799231

[B65] Pennell DJ Sechtem UP Higgins CB Manning WJ Pohost GM Rademakers FE van Rossum AC Shaw LJ Yucel EK Clinical indications for cardiovascular magnetic resonance (CMR): Consensus Panel report J Cardiovasc Magn Reson 2004 6 727 65 10.1081/JCMR-200038581 15646878

[B66] Fallah-Rad N Walker JR Wassef A Lytwyn M Bohonis S Fang T Tian G Kirkpatrick ID Singal PK Krahn M The utility of cardiac biomarkers, tissue velocity and strain imaging, and cardiac magnetic resonance imaging in predicting early left ventricular dysfunction in patients with human epidermal growth factor receptor II-positive breast cancer treated with adjuvant trastuzumab therapy J Am Coll Cardiol 2011 57 2263 70 10.1016/j.jacc.2010.11.063 21616287

[B67] Banchs J Jefferies JL Plana JC Hundley WG Imaging for cardiotoxicity in cancer patients Tex Heart Inst J 2011 38 268 9 21720469 PMC3113123

[B68] Adams MJ Hardenbergh PH Constine LS Lipshultz SE Radiation-associated cardiovascular disease Crit Rev Oncol Hematol 2003 45 55 75 10.1016/S1040-8428(01)00227-X 12482572

[B69] Schellong G Riepenhausen M Bruch C Kotthoff S Vogt J Bolling T Dieckmann K Potter R Heinecke A Bramswig J Dorffel W Late valvular and other cardiac diseases after different doses of mediastinal radiotherapy for Hodgkin disease in children and adolescents: report from the longitudinal GPOH follow-up project of the German-Austrian DAL-HD studies Pediatr Blood Cancer 2010 55 1145 52 10.1002/pbc.22664 20734400

[B70] Galper SL Yu JB Mauch PM Strasser JF Silver B Lacasce A Marcus KJ Stevenson MA Chen MH Ng AK Clinically significant cardiac disease in patients with Hodgkin lymphoma treated with mediastinal irradiation Blood 2011 117 412 8 10.1182/blood-2010-06-291328 20858859

[B71] Chiles C Woodard PK Gutierrez FR Link KM Metastatic involvement of the heart and pericardium: CT and MR imaging Radiographics 2001 21 439 49 11259706 10.1148/radiographics.21.2.g01mr15439

[B72] Abraham KP Reddy V Gattuso P Neoplasms metastatic to the heart: review of 3314 consecutive autopsies Am J Cardiovasc Pathol 1990 3 195 8 2095826

[B73] Mason JW Bristow MR Billingham ME Daniels JR Invasive and noninvasive methods of assessing adriamycin cardiotoxic effects in man: superiority of histopathologic assessment using endomyocardial biopsy Cancer Treat Rep 1978 62 857 64 667859

[B74] Thompson RC Canby RC Lojeski EW Ratner AV Fallon JT Pohost GM Adriamycin cardiotoxicity and proton nuclear magnetic resonance relaxation properties Am Heart J 1987 113 1444 9 10.1016/0002-8703(87)90660-0 3591613

[B75] Cottin Y Ribuot C Maupoil V Godin D Arnould L Brunotte F Rochette L Early incidence of adriamycin treatment on cardiac parameters in the rat Can J Physiol Pharmacol 1994 72 140 5 10.1139/y94-022 8050054

[B76] Wassmuth R Lentzsch S Erdbruegger U Schulz-Menger J Doerken B Dietz R Friedrich MG Subclinical cardiotoxic effects of anthracyclines as assessed by magnetic resonance imaging-a pilot study Am Heart J 2001 141 1007 13 10.1067/mhj.2001.115436 11376317

[B77] Lightfoot JC D’Agostino RB Jr Hamilton CA Jordan J Torti FM Kock ND Workman S Hundley WG Novel approach to early detection of doxorubicin cardiotoxicity by gadolinium-enhanced cardiovascular magnetic resonance imaging in an experimental model Circ Cardiovasc Imaging 2010 3 550 8 10.1161/CIRCIMAGING.109.918540 20622140 PMC3068484

[B78] Broberg CS Chugh SS Conklin C Sahn DJ Jerosch-Herold M Quantification of diffuse myocardial fibrosis and its association with myocardial dysfunction in congenital heart disease Circ Cardiovasc Imaging 2010 3 727 34 10.1161/CIRCIMAGING.108.842096 20855860 PMC3048790

[B79] Jerosch-Herold M Sheridan DC Kushner JD Nauman D Burgess D Dutton D Alharethi R Li D Hershberger RE Cardiac magnetic resonance imaging of myocardial contrast uptake and blood flow in patients affected with idiopathic or familial dilated cardiomyopathy Am J Physiol Heart Circ Physiol 2008 295 H1234 42 10.1152/ajpheart.00429.2008 18660445 PMC2544489

[B80] Schelbert EB Testa SM Meier CG Ceyrolles WJ Levenson JE Blair AJ Kellman P Jones BL Ludwig DR Schwartzman D Myocardial extravascular extracellular volume fraction measurement by gadolinium cardiovascular magnetic resonance in humans: slow infusion versus bolus J Cardiovasc Magn Reson 2011 13 16 10.1186/1532-429X-13-16 21375743 PMC3059279

[B81] Messroghli DR Greiser A Fröhlich M Dietz R Schulz-Menger J Optimization and validation of a fully-integrated pulse sequence for modified look-locker inversion-recovery (MOLLI) T1 mapping of the heart J Magn Reson Imaging 2007 26 1081 6 10.1002/jmri.21119 17896383

[B82] Messroghli DR Plein S Higgins DM Walters K Jones TR Ridgway JP Sivananthan MU Human myocardium: single-breath-hold MR T1 mapping with high spatial resolution–reproducibility study Radiology 2006 238 1004 12 10.1148/radiol.2382041903 16424239

[B83] Messroghli DR Radjenovic A Kozerke S Higgins DM Sivananthan MU Ridgway JP Modified Look-Locker inversion recovery (MOLLI) for high-resolution T1 mapping of the heart Magn Reson Med 2004 52 141 6 10.1002/mrm.20110 15236377

[B84] Flett AS Hayward MP Ashworth MT Hansen MS Taylor AM Elliott PM McGregor C Moon JC Equilibrium contrast cardiovascular magnetic resonance for the measurement of diffuse myocardial fibrosis: preliminary validation in humans Circulation 2010 122 138 44 10.1161/CIRCULATIONAHA.109.930636 20585010

[B85] Wong TC Piehler K Puntil KS Moguillansky D Meier CG Lacomis JM Kellman P Cook SC Schwartzman DS Simon MA Effectiveness of late gadolinium enhancement to improve outcomes prediction in patients referred for cardiovascular magnetic resonance after echocardiography J Cardiovasc Magn Reson 2013 15 6 10.1186/1532-429X-15-6 23324403 PMC3599652

[B86] Schwartz RG McKenzie WB Alexander J Sager P D’Souza A Manatunga A Schwartz PE Berger HJ Setaro J Surkin L Congestive heart failure and left ventricular dysfunction complicating doxorubicin therapy. Seven-year experience using serial radionuclide angiocardiography Am J Med 1987 82 1109 18 10.1016/0002-9343(87)90212-9 3605130

[B87] Jones AL Barlow M Barrett-Lee PJ Canney PA Gilmour IM Robb SD Plummer CJ Wardley AM Verrill MW Management of cardiac health in trastuzumab-treated patients with breast cancer: updated United Kingdom National Cancer Research Institute recommendations for monitoring Br J Cancer 2009 100 684 92 10.1038/sj.bjc.6604909 19259090 PMC2653760

[B88] Bovelli D Plataniotis G Roila F Cardiotoxicity of chemotherapeutic agents and radiotherapy-related heart disease: ESMO Clinical Practice Guidelines Ann Oncol 2010 21 Suppl 5 v277 82 20555097 10.1093/annonc/mdq200

[B89] Grothues F Smith GC Moon JC Bellenger NG Collins P Klein HU Pennell DJ Comparison of interstudy reproducibility of cardiovascular magnetic resonance with two-dimensional echocardiography in normal subjects and in patients with heart failure or left ventricular hypertrophy Am J Cardiol 2002 90 29 34 10.1016/S0002-9149(02)02381-0 12088775

[B90] Pattynama PM Lamb HJ van der Velde EA van der Wall EE de Roos A Left ventricular measurements with cine and spin-echo MR imaging: a study of reproducibility with variance component analysis Radiology 1993 187 261 8 8451425 10.1148/radiology.187.1.8451425

[B91] Stoodley PW Richards DA Hui R Boyd A Harnett PR Meikle SR Clarke J Thomas L Two-dimensional myocardial strain imaging detects changes in left ventricular systolic function immediately after anthracycline chemotherapy Eur J Echocardiogr 2011 12 945 52 10.1093/ejechocard/jer187 21965152

[B92] Motoki H Borowski AG Shrestha K Troughton RW Tang WH Thomas JD Klein AL Incremental prognostic value of assessing left ventricular myocardial mechanics in patients with chronic systolic heart failure J Am Coll Cardiol 2012 60 2074 81 10.1016/j.jacc.2012.07.047 23083779

[B93] Cho GY Marwick TH Kim HS Kim MK Hong KS Oh DJ Global 2-dimensional strain as a new prognosticator in patients with heart failure J Am Coll Cardiol 2009 54 618 24 10.1016/j.jacc.2009.04.061 19660692

[B94] Bellavia D Abraham RS Pellikka PA Dispenzieri A Burnett JC Jr Al-Zahrani GB Green TD Manske MK Gertz MA Miller FA Jr Abraham TP Utility of Doppler myocardial imaging, cardiac biomarkers, and clonal immunoglobulin genes to assess left ventricular performance and stratify risk following peripheral blood stem cell transplantation in patients with systemic light chain amyloidosis (Al) J Am Soc Echocardiogr 2011 24 444 54 10.1016/j.echo.2011.01.003 21315556 PMC3065954

[B95] Saito M Okayama H Yoshii T Higashi H Morioka H Hiasa G Sumimoto T Inaba S Nishimura K Inoue K Clinical significance of global two-dimensional strain as a surrogate parameter of myocardial fibrosis and cardiac events in patients with hypertrophic cardiomyopathy Eur Heart J Cardiovasc Imaging 2012 13 617 23 10.1093/ejechocard/jer318 22271116

[B96] Drafts BC Twomley KM D’Agostino RB Lawrence JA Avis NE Ellis LR Thohan V Jordan J Melin SA Torti FM Low to moderate dose anthracyline-based chemotherapy is associated with early noninvasive imaging evidence of subclinical cardiovascular disease JACC Cardiovasc Imaging 2012 Accepted for publication 10.1016/j.jcmg.2012.11.017 PMC3745801 23643285

[B97] Pituskin E Haykowsky M Mackey JR Thompson RB Ezekowitz J Koshman S Oudit G Chow K Pagano JJ Paterson I Rationale and design of the Multidisciplinary Approach to Novel Therapies in Cardiology Oncology Research Trial (MANTICORE 101–Breast): a randomized, placebo-controlled trial to determine if conventional heart failure pharmacotherapy can prevent trastuzumab-mediated left ventricular remodeling among patients with HER2+ early breast cancer using cardiac MRI BMC Cancer 2011 11 318 10.1186/1471-2407-11-318 21794114 PMC3171383

[B98] McGill LA Ismail TF Nielles-Vallespin S Ferreira P Scott AD Roughton M Kilner PJ Ho SY McCarthy KP Gatehouse PD Reproducibility of in-vivo diffusion tensor cardiovascular magnetic resonance in hypertrophic cardiomyopathy J Cardiovasc Magn Reson 2012 14 86 10.1186/1532-429X-14-86 23259835 PMC3551746

[B99] Rider OJ Francis JM Tyler D Byrne J Clarke K Neubauer S **Effects of weight loss on myocardial energetics and diastolic function in obesity** Int J Cardiovasc Imaging 2013 29 5 1043 50 10.1007/s10554-012-0174-6 23269470

[B100] Saigal CS Gore JL Krupski TL Hanley J Schonlau M Litwin MS Androgen deprivation therapy increases cardiovascular morbidity in men with prostate cancer Cancer 2007 110 1493 500 10.1002/cncr.22933 17657815

[B101] Chu TF Rupnick MA Kerkela R Dallabrida SM Zurakowski D Nguyen L Woulfe K Pravda E Cassiola F Desai J Cardiotoxicity associated with tyrosine kinase inhibitor sunitinib Lancet 2007 370 2011 9 10.1016/S0140-6736(07)61865-0 18083403 PMC2643085

[B102] Escudier B Eisen T Stadler WM Szczylik C Oudard S Staehler M Negrier S Chevreau C Desai AA Rolland F Sorafenib for treatment of renal cell carcinoma: final efficacy and safety results of the phase III treatment approaches in renal cancer global evaluation trial J Clin Oncol 2009 27 3312 8 10.1200/JCO.2008.19.5511 19451442

[B103] Miller K Wang M Gralow J Dickler M Cobleigh M Perez EA Shenkier T Cella D Davidson NE Paclitaxel plus bevacizumab versus paclitaxel alone for metastatic breast cancer N Engl J Med 2007 357 2666 76 10.1056/NEJMoa072113 18160686

[B104] Eckman DM Stacey RB Rowe R D. Agostino RJ Kock ND Sane DC Torti FM Yeboah J Workman S Lane KS Hundley WG Weekly Doxorubicin increases coronary arteriolar wall and adventitial thickness PLoS One 2013 8 e57554 10.1371/journal.pone.0057554 23437398 PMC3578811

[B105] Rerkpattanapipat P Hundley WG Link KM Brubaker PH Hamilton CA Darty SN Morgan TM Kitzman DW Relation of aortic distensibility determined by magnetic resonance imaging in patients > or =60 years of age to systolic heart failure and exercise capacity Am J Cardiol 2002 90 1221 5 10.1016/S0002-9149(02)02838-2 12450602

[B106] Meeske K Smith AW Alfano CM McGregor BA McTiernan A Baumgartner KB Malone KE Reeve BB Ballard-Barbash R Bernstein L Fatigue in breast cancer survivors two to five years post diagnosis: a HEAL Study report Qual Life Res 2007 16 947 60 10.1007/s11136-007-9215-3 17457697

[B107] Pater JL Loeb M Nonanatomic prognostic factors in carcinoma of the lung: a multivariate analysis Cancer 1982 50 326 31 10.1002/1097-0142(19820715)50:2<326::AID-CNCR2820500227>3.0.CO;2-G 7083139

[B108] Ringdal GI Gotestam KG Kaasa S Kvinnsland S Ringdal K Prognostic factors and survival in a heterogeneous sample of cancer patients Br J Cancer 1996 73 1594 9 10.1038/bjc.1996.300 8664136 PMC2074532

[B109] Andersen C Rørth M Ejlertsen B Stage M Møller T Midtgaard J Quist M Bloomquist K Adamsen L **The effects of a six-week supervised multimodal exercise intervention during chemotherapy on cancer-related fatigue** Eur J Oncol Nurs 2013 17 3 331 9 10.1016/j.ejon.2012.09.003 23084254

[B110] Holmes MD Chen WY Feskanich D Kroenke CH Colditz GA Physical activity and survival after breast cancer diagnosis JAMA 2005 293 2479 86 10.1001/jama.293.20.2479 15914748

[B111] Meyerhardt JA Giovannucci EL Holmes MD Chan AT Chan JA Colditz GA Fuchs CS Physical activity and survival after colorectal cancer diagnosis J Clin Oncol 2006 24 3527 34 10.1200/JCO.2006.06.0855 16822844

[B112] Hundley WG Bayram E Hamilton CA Hamilton EA Morgan TM Darty SN Stewart KP Link KM Herrington DM Kitzman DW Leg flow-mediated arterial dilation in elderly patients with heart failure and normal left ventricular ejection fraction Am J Physiol Heart Circ Physiol 2007 292 H1427 34 17085542 10.1152/ajpheart.00567.2006

[B113] Puntawangkoon C Kitzman DW Kritchevsky SB Hamilton CA Nicklas B Leng X Brubaker PH Hundley WG Reduced peripheral arterial blood flow with preserved cardiac output during submaximal bicycle exercise in elderly heart failure J Cardiovasc Magn Reson 2009 11 48 10.1186/1532-429X-11-48 19922666 PMC2789719

[B114] Gilliam LA Ferreira LF Bruton JD Moylan JS Westerblad H St Clair DK Reid MB Doxorubicin acts through tumor necrosis factor receptor subtype 1 to cause dysfunction of murine skeletal muscle J Appl Physiol 2009 107 1935 42 10.1152/japplphysiol.00776.2009 19779154 PMC2793196

[B115] Gilliam LA Moylan JS Ann Callahan L Sumandea MP Reid MB Doxorubicin causes diaphragm weakness in murine models of cancer chemotherapy Muscle Nerve 2011 43 94 102 10.1002/mus.21809 21171100 PMC3057655

[B116] Doroshow JH Tallent C Schechter JE Ultrastructural features of Adriamycin-induced skeletal and cardiac muscle toxicity Am J Pathol 1985 118 288 97 3970141 PMC1887878

[B117] Jacobi B Bongartz G Partovi S Schulte AC Aschwanden M Lumsden AB Davies MG Loebe M Noon GP Karimi S Skeletal muscle BOLD MRI: from underlying physiological concepts to its usefulness in clinical conditions J Magn Reson Imaging 2012 35 1253 65 10.1002/jmri.23536 22588992

[B118] Ledermann HP Heidecker HG Schulte AC Thalhammer C Aschwanden M Jaeger KA Scheffler K Bilecen D Calf muscles imaged at BOLD MR: correlation with TcPO2 and flowmetry measurements during ischemia and reactive hyperemia–initial experience Radiology 2006 241 477 84 10.1148/radiol.2412050701 16982813

